# The therapeutic potential of circular RNA in triple-negative breast cancer

**DOI:** 10.20517/cdr.2023.141

**Published:** 2024-04-23

**Authors:** Aiqi Xu, Lewei Zhu, Chengcai Yao, Wen Zhou, Ziyun Guan

**Affiliations:** ^1^Department of Breast Oncology, School of Medicine, South China University of Technology, Guangzhou 510006, Guangdong, China.; ^2^Department of Breast Surgery, The First People’s Hospital of Foshan, Foshan 528000, Guangdong, China.; ^3^The Sixth Affiliated Hospital, School of Medicine, South China University of Technology, Foshan 528200, Guangdong, China.; ^#^Authors contributed equally.

**Keywords:** Triple-negative breast cancer, circular RNA, non-coding RNA

## Abstract

Triple-negative breast cancer (TNBC) is among the most aggressive subtypes of the disease that does not express estrogen receptor, progesterone receptor, and human epidermal growth factor receptor 2. Circular RNAs (circRNAs) are a type of non-coding RNA with a circular shape formed by non-standard splicing or reverse splicing. Numerous circRNAs exhibit abnormal expression in various malignancies, showing their critical role in the emergence and growth of tumors. Recent studies have shown evidence supporting the idea that certain circRNAs regulate the proliferation and metastasis of TNBC. In addition, circRNAs alter metabolism and the immune microenvironment to promote or inhibit the development of TNBC. Notably, circRNAs may affect the efficacy of clinical drug therapy, serve as therapeutic targets, and be used as molecular biomarkers in the future. Herein, we will first summarize the biogenesis and function of circRNAs. Then, we will explain current research on circRNAs related to TNBC and their potential to serve as therapeutic targets or biomarkers for future drug development, providing a new direction and idea for TNBC therapy.

## INTRODUCTION

The latest research report indicates that breast cancer is now the most prevalent form of cancer in the world, with the highest incidence among all major cancers^[1]^. According to its histological characteristics and expression of estrogen receptor (ER), progesterone receptor (PR) and human epidermal growth factor receptor 2 (HER2), breast cancer can be subdivided into luminal A, luminal B, HER2 overexpression, and triple-negative breast cancer (TNBC), respectively^[2,3]^. The incidence of TNBC ranges from 15% to 20%^[4]^ and it is difficult to identify the potential therapeutic targets. Thus, it leads to an increased risk of recurrence and metastasis and the patients have poor prognosis and shorter overall survival^[5]^. Investigation into the detailed molecular pathogenesis of TNBC and the development of potential therapeutic targets are important tasks in order to improve the prognosis more effectively and prolong the survival time of TNBC patients.

Circular RNAs (circRNAs) have recently gained significant attention as they are involved in various diseases, including human cancers^[6]^ such as TNBC. Many studies suggested that circRNAs are strongly associated with the occurrence and development of tumors. They are non-coding RNAs that have been recently discovered and are formed by reverse splicing the precursor messenger RNAs (mRNAs). Compared with linear RNA, circRNAs do not have a 5 'end cap and a 3' end polyA tail which is more stable and not easily degraded by nuclease^[7,8]^. Its main functions include microRNA (miRNA) sponging^[9]^, RNA binding protein (RBP) binding^[10]^, and serving as a translation template^[11]^. Studies have indicated that circRNAs have the capability to regulate downstream genes and contribute to tumor proliferation, invasion, and metastasis. Therefore, identifying the specific role that circRNAs play in tumor development and occurrence is of great importance.

The first discovery of circRNA in eukaryotic cells was made through electron microscopy in 1979^[12]^. However, due to the scientific limitations at that time, circRNA was considered as a product of abnormal splicing events^[13]^. With the advancement of next-generation sequencing technology and bioinformatic analysis tools, a large number of circRNAs have been identified in eukaryotic genome and transcriptome^[14]^. This identification implies that circRNAs are not accidental by-products, but normal components of the body that are widely expressed in eukaryotes, including humans^[15]^. Therefore, scientists began to conduct detailed research on various functions served by circRNAs. Recently, an increasing number of circRNAs have been reported to be differentially expressed in breast cancer. These aberrantly expressed circRNAs mediate a range of tumorigenic processes, including cell proliferation, metastasis, apoptosis, and cell metabolism^[16]^. Hence, we summarized current information on abnormal expression of specific circRNAs that are implicated in TNBC development, followed by current clinical applications and potential therapeutic utility of circRNAs. It is clear that circRNA holds promise as a future therapeutic target^[17]^.

## FORMATION AND TYPES OF CircRNAs

**Figure 1:**
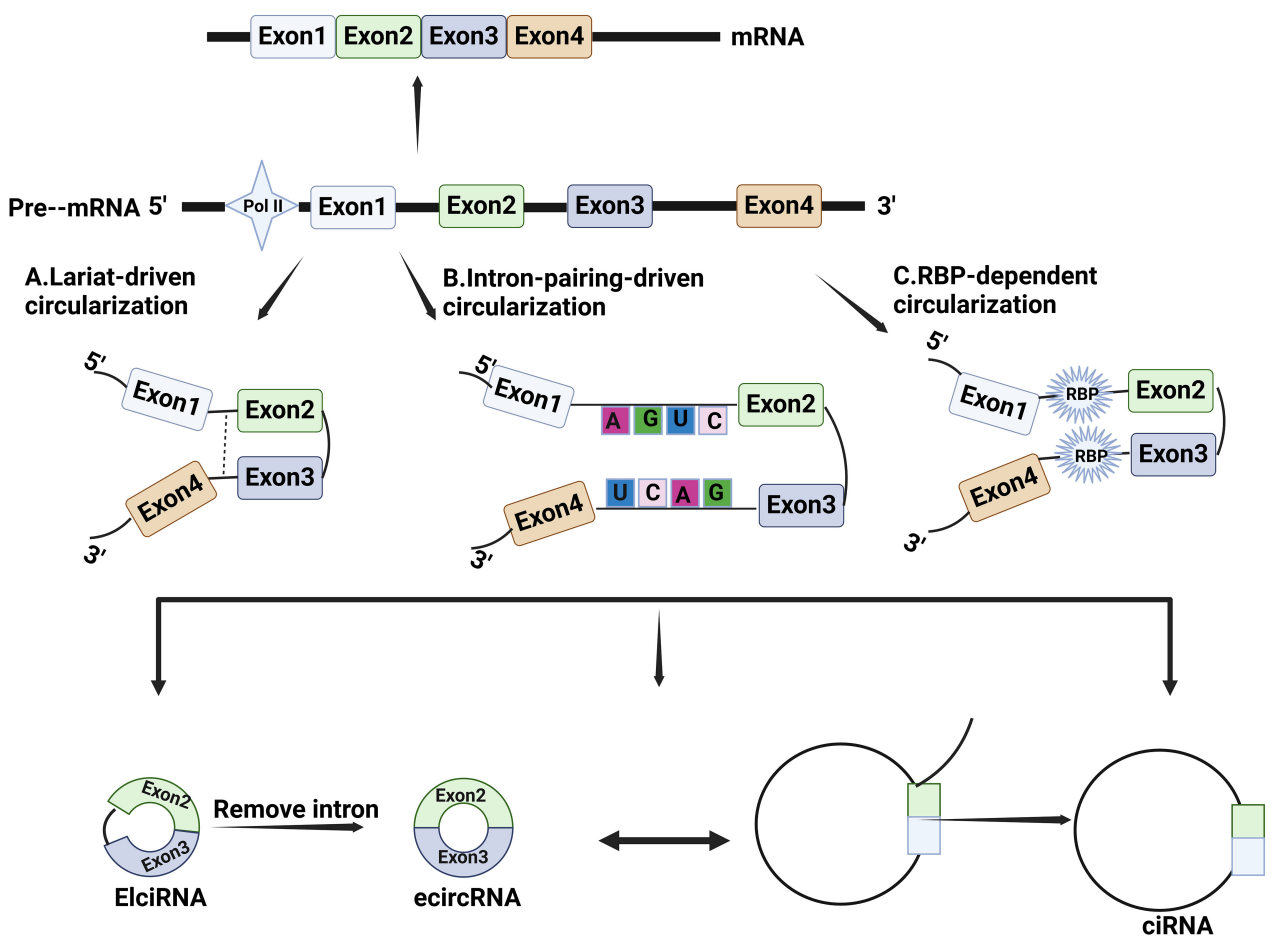
Biosynthetic mechanisms for circRNAs. (A) Lariat-driven circularization: lariat causes exons or introns that were originally far apart to come closer; (B) Intron pairing-driven circularization: complementary sequences in introns promote circRNA circularization by base pairing to bring the 3' splice acceptor site and the 5' splice donor site closer together in spatial conformation; (C) RBP-dependent circularization: RBPs bind to intronic sequences or specific motifs within flanking introns near the splice site and indirectly bridge the distance between upstream and downstream exons. RBP: RNA binding protein.

The mechanism for circRNA formation is still not clear. It is generally accepted that they are derived from precursor mRNAs (pre-mRNAs), which are closed-loop molecules formed by back splicing^[8]^. Depending on their composition and synthetic mechanism, circRNAs can be categorized into three main groups: EciRNA, EIciRNA, and ciRNA^[18]^. First, EciRNAs are derived entirely from the exons of parental genes. In the process of forming EciRNA, a splice donor downstream of the 5' splice site is attached to a splice acceptor upstream of the 3' splice site, which is called back-splicing^[15]^. This molecular event produces a circular format of RNA with a 3'-5' phosphodiester bond at the back-splicing junction (BSJ) site. EciRNA is the most common type of circRNA and is mainly located in the cytoplasm^[19]^. Depending on the distribution of specific organelles, these circRNAs in the cytoplasm perform different functions. Second, EIciRNAs are circRNAs resulting from the retention of introns located in the 5' donor and the 3' acceptor on the pre-mRNAs. These circRNAs consist of both exons and introns. It is predominately located in the nucleus^[19]^. This means they may provide transcript and splicing functions^[20,21]^. Finally, ciRNAs are formed only by the head-to-tail joining of intronic sequences and located primarily in the nucleus^[19]^. The mechanism of formation of the first two circRNAs is relatively similar. There are currently three mechanism hypotheses for the origination of EciRNAs and EIciRNAs: intron pairing-driven circularization, RNA binding protein (RBP)-dependent circularization, and lariat-driven circularization^[22]^ [Figure 1]. For intron pairing-driven circularization, complementary sequences in introns promote circRNA circularization by base pairing to bring the 3' splice acceptor site and the 5' splice donor site closer together in spatial conformation. Sequences known to promote intron circularization are Alu repeat elements^[23]^. For RBP-dependent circularization, it can promote circRNA formation by binding to intronic sequences or specific motifs within flanking introns near the splice site and indirectly bridging the distance between upstream and downstream exons, thus promoting loop formation^[24]^. Representative RBPs include FUS^[25]^ and MBL^[26]^. For lariat-driven circularization, partial folding of exons may occur during the transcription process of pre-mRNA, leading to the formation of lariat. These folds cause exons or introns that were originally far apart to come closer, thereby promoting the formation of circRNA^[27]^. CiRNA formation is less similar to circRNAs containing exons, in that it relies on undegraded lariats that contain important sequences such as GU repeats at the 5' splice site and C elements enriched close to the branch site, allowing the 5' end site to form a 2'-5' linkage with the 2'-OH to form circRNA^[28]^. Most circRNAs belong to the exon-containing types, and their conservation is higher than other types of circRNAs.

The vast majority of circRNAs have corresponding linear parental genes in organisms, which makes it difficult to be distinguished easily. Currently, the main approach is first to enrich circRNAs based on their unique stable structure by digesting total RNA with RNase R, and then to identify specific circRNAs through the detection of specific junction sequences by RNA high-throughput sequencing^[29]^. Although these methods can identify many BSJ sequences, their detection sensitivity is low once the BSJ sequence is very short. In recent years, some emerging long-read sequencing technologies have gradually developed, such as PacBio and Oxford Nanopore, which can better distinguish circRNA from its corresponding linear transcripts^[30]^. Furthermore, Chiang *et al*. have recently developed a FL-circAS nanopore long-read sequencing technology, which can decipher circRNAs from the aspects of their expression, formation, and function. This technology will promote the further development of circRNA research^[31]^.

## The FUNCTION OF CIRCULAR RNA IN TNBC

### Sponging miRNA

In accordance with the base complementary pairing principle, miRNA inhibits or facilitates mRNA translation by binding to non-translational regions in target genes^[32]^. RNA that can competitively bind to common miRNAs to inhibit their activity on target genes is known as competitive endogenous RNA (ceRNA)^[33]^. Research has revealed that circRNAs carry a large amount of miRNA-responsive sequences, which can serve as effective ceRNAs. They bind to miRNA to adsorb it and effectively prevent its binding to the target genes, thereby providing regulatory effects on target genes^[34]^.

Research on circRNAs in TNBC primarily concentrated on understanding the function of miRNA sponging. For example, CiRS-7 may be the most distinctive circRNA, containing over 70 conserved binding sites of miR-7, and has a stable expression in many tumors including TNBC^[35]^. Recent research shows that CiRS-7 also has miR-1299 binding sites besides miR-7. Experiments identified that downregulation of CiRS-7 expression inhibits migration and invasion of TNBC cells *in vitro* and suppresses their metastasis to the liver and lung *in vivo*. CiRS-7 regulates the expression of matrix metalloproteinases (MMP) family members through sponging miR-1299^[36]^. Li found that circCRIM1 is upregulated in TNBC, which enhanced the expression of glycosylation hydrolase O-GlcNAcase (OGA) through miR-503-5p to regulate lipid metabolism and thus promote TNBC development^[37]^. Similarly, CircWHS promotes TNBC progression by regulating AKT3 expression through miR-212-5p to enhance glycolytic capacity^[38]^. In terms of drug resistance, it was found that CircEGFR regulates expression of the epidermal growth factor receptor (EGFR) through miR-1299, which in turn promotes TNBC progression and resistance to trastuzumab and pertuzumab in combination with taxanes (THP) therapy^[39]^. In addition, CircDUSP1 enhances expression of the disheveled binding antagonist of beta-catenin 2 (DACT2) via miR-761, thereby promoting TNBC sensitivity to paclitaxel^[40]^; circUBE2D2 silences miR-512-3p to enhance CDCA3 to promote doxorubicin resistance in TNBC^[41]^; and CircNCOR1 mediates TNBC radiotherapy resistance via hsa-miR-638^[42]^. At the immune microenvironment level, circFGFR4 promotes TNBC immune evasion and resistance to PD-1 immunotherapy through miR-185-5p^[43]^. A number of circRNA-miRNA networks were summarized in Table 1.

**Table 1:** Summary of miRNAs currently bind to circRNAs in TNBC

**CircRNAs**	**Expression in TNBC**	**MiRNAs**	**Targets**	**Functions**	**Ref.**
CircCRIM1	High	miR-503-5p	OGA/FBP1	Increases glycosylation hydrolase OGA and decreases FBP1	[37]
CircEGFR	High	miR-1299	EGFR	Regulates malignant progression of TNBC by controlling EGFR through miR-1299, leading to THP drug-resistant	[39]
CircNCOR1	Low	miR-638	CDK2	CircNCOR1 binds to hsa-miR-638 and targets CDK2 to regulate TNBC radio-sensitivity	[42]
CircFGFR4	High	miR-185-5p	CXCR4	CircFGFR4 exerts an impact on immune evasion and resistance to PD-1 immunotherapy through miR-185-5p/CXCR4 axis in TNBC	[43]
CircDUSP1	Low	miR-761	DACT2	Reduces the suppression of DACT2 expression by miR-761 and thus increases the sensitivity of TNBC cells to paclitaxel	[40]
Circ-TRIO	High	miR-432-5p	CCDC58	Circ-TRIO may regulate CCDC58 expression by combining with miR-432-5p	[44]
CircRAD54L2	High	miR-888	PDK1	Regulates PDK1 expression by sponging the miR-888 family and PDK1	[45]
Circ0004676	High	miR-377-3p	E2F6/PNO1	Circ0004676 increases E2F6 and its target PNO1 expression by sponging miR-377-3p	[46]
CircCSNK1G1	High	miR-28-5p	LDHA	Circ-CSNK1G1 represses miR-28-5p and positively regulates LDHA expression	[47]
CircUBR5	High	miR-1179	UBR5	CircUBR5 upregulates UBR5 expression by suppressing miR-1179	[48]
CircDHDDS	High	miR-362-3p	DDX5	CircDHDDS accelerates TNBC by upregulating DDX5 via miR-362-3p adaptation	[49]
CircPRKCI	High	miR-545-3p	WBP2 and PI3K/AKT	CircPRKCI acts as miR-545-3p sponge to regulate WBP2 and AKT phosphorylation	[50]
CircPTK2	Low	miR-136	NFBI and AKT/PI3K	CircPTK2 serves as miR-136 sponge and direct regulator of NFBI and AKT/PI3K pathways	[51]
CircUBAP2	High	miR-300	ASF1B and PI3K/AKT/mTOR	CircUBAP2 upregulates ASF1B by miR-300, which triggers (PAM) signaling to increase DDP resistance in TNBC.	[52]
CircFAM64A	High	miR-149-5p	CDT1	CircFAM64A sponges miR-149-5p to increase CDT1 expression and promote cellular processes in TNBC	[53]
CircCD44	High	miR-502-5p	KRAS and IGF2BP2/Myc	CircCD44 contributes to TNBC cell proliferation, migration, invasion, and tumorigenesis, in part through miR-502-5p sponge activity and interaction with IGF2BP2	[54]
Circ-PDCD11	High	miR-432-5p	LDHA	By sponging up miR-432-5p, circPDCD11 enhances LDHA expression	[55]
Circ-ERBB2	High	miR-136-5p	PDK4	As a result of miR-136-5p inhibition by circ-ERBB2, PDK4 expression is increased	[56]
CircWHSC1	High	miR-212-5p	AKT3	CircWHSC1 promotes malignancy and glycolysis in TNBC cells by sequestering miR-212-5p.	[38]
CircWAC	High	miR-142	WWP1 and PI3K/AKT	CircWAC sponges miR-142 to elevate WWP1 expression and activate PI3K/AKT pathway	[57]
CircNR3C2	Low	miR-513a-3p	HRD1/Vimentin	Overexpression of circNR3C2 significantly improves the tumor suppressive effects of HRD1 by sponging miR-513a-3p	[58]
CircPSMA1	High	miR-637	Akt1/β-catenin (cyclin D1)	CircPSMA1 functions as a promoter of tumor growth through the circPSMA1/miR-637/Akt1-β-catenin (cyclin D1) regulatory pathway	[59]
CircBACH2	High	miR-186-5p /miR-548c-3p	CXCR4	The expression of CXCR4 is increased, resulting from the sponging of miR-186-5p and miR-548c-3p by ircBACH2	[60]
Circ_102229	High	miR-152-3p	PFTK1	Hsa_circ_102229 could regulate the expression of PFTK1 by targeting miR-152-3p	[61]
CircUBE2D2	High	miR-512-3p	CDCA3	CircUBE2D2 facilitates the advancement of TNBC and increases resistance to doxorubicin by functioning as a miR-512-3p sponge, which results in elevated CDCA3 expression	[41]
Circ0000199	High	miR-613 and miR-206	PI3K/Akt/mTOR	Hsa_circ_0000199 improves sensitivity to chemotherapy in TNBC by promoting the expression of miR-206/miR-613 and deactivating PI3K/Akt/mTOR signaling pathway	[62]
CircIFI30	High	miR-520b-3p	CD44	CircIFI30 sponges miR-520b-3p, thus releasing the CD44 expression	[63]
CircPGAP3	High	miR-330-3p	Myc	Circ-PGAP3 promotes the development of TNBC cells by inhibiting miR-330-3p through its sponging function, leading to increased gene expression of the proto-oncogene Myc	[64]
CircFBXW7	Low	miR-197-3p	FBXW7	*FBXW7* gene sponges miR-197-3p and produces FBXW7-185aa protein to inhibit TNBC development by increasing FBXW7 expression	[11]
CircPLK1	High	miR-296-5p	PLK1	CircPLK1 may be a target of miR-296-5p for the regulation of PLK1 expression	[65]
CircKIF4A	High	miR-637	STAT3	CircKIF4A regulates STAT3 expression levels through miR-375	[66]
CircEPSTI1	High	miR-4753 miR-6809	BCL11A	CircEPSTI1 acts as a sponge for miR-4753 and miR-6809, which regulate the expression of BCL11A	[67]

TNBC: Triple-negative breast cancer; OGA: O-GlcNAcase; EGFR: epidermal growth factor receptor; THP: trastuzumab and pertuzumab in combination with taxanes; DACT2: disheveled binding antagonist of beta-catenin 2; LDHA: lactate dehydrogenase A.

### Binding to proteins

Results from a cross-linked immunoprecipitation experiment showed that circRNA interacts with various proteins^[68]^. The first study to demonstrate the function of protein binding was conducted on the splicing factor protein gene encoding muscle blindness (MBL) in *Drosophila melanogaster*, a homolog of human muscle protein 1. The gene produces circMBL in both Drosophila and humans, with the binding sites of MBL and MBNL1, respectively^[69]^. Similar to circMBL, other circRNAs in breast cancer also function by connecting with their target proteins. For example, circKIF4A stabilizes the mRNA expression of SDC1 by attaching to EIF4A, triggers the c-src/FAK signaling pathway, and leads to the advancement of disease in TNBC^[70]^. Mass spectrometry and RNA Binding Protein Immunoprecipitation experiments demonstrated that circSNX25 binds to COPB1(Coatomer Protein Complex, Subunit Beta 1) to promote the malignant progression of TNBC, bringing extremely poor prognosis^[71]^. The above two examples of circRNAs are intended to demonstrate their functions in binding to proteins. Additionally, circRNAs have the ability to facilitate mRNA translation. Circ_0076611 is a circRNA produced by MALAT1-dependent production that binds to many transcripts, including the mRNAs for MYC and VEGFA. It can affect the cell cycle and promote cell proliferation by facilitating the binding of MYC and VEGFA mRNAs to translation initiation factors and elevating their protein expression, leading to TNBC progression^[72]^. It also functions as a protein scaffold. For example, circEIF3H can directly combine with IGF2BP and HuR proteins to form the circEIF3H-IGF2BP2-HuR scaffolding complex, which in turn is inextricably linked to mRNA stability. In this case, circEIF3H promotes TNBC progression by stabilizing downstream HSPD1/RBM8A/G3BP1 mRNA expression in an indirect manner^[73]^.

### Encoding proteins

Recent research found that circRNA encodes proteins, which changes the traditional recognition that circRNA is non-coding RNA. Either the internal ribosome entry site (IRES)^[74]^ or the m^6^ 5' “untranslated region (UTR)” can be used for cell-independent translation of circRNA^[75]^. The protein encoded by circRNA can activate some downstream signal pathways to promote the occurrence of TNBC. CircFBXW7 can sponge miR-197-3p and encode a 185-aa protein, thus inhibiting malignant progression in TNBC^[11]^. CircCAPG produces a polypeptide known as CAPG-171aa. This polypeptide promotes cancer growth by inhibiting the binding between serine/threonine kinase 38 (STK38) and SMAD-specific E3 ubiquitin protein ligase 1 (SMURF1), which prevents MEKK2 from undergoing ubiquitination and proteasomal degradation^[76]^. Circ-EIF6 exerts pro-oncogenic effects on TNBC through its encoded peptide EIF6-224aa, which decreases the ubiquitinated degradation of the oncogene MYH9, thereby increasing its expression to activate the downstream Wnt/beta-catenin signaling pathway^[77]^. CircSEMA4B, which is significantly downregulated in TNBC tissues and cell lines, encodes a novel protein called SEMA4B. Both circSEMA4B and SEMA4B inhibit TNBC proliferation and migration *in vitro* and *in vivo*^[78]^. In addition, a study has shown that circHER2 is present in TNBC and encodes the novel protein HER2-103. This protein targets the HER2-targeting drug Pertuzumab, which has been commonly used in clinical treatment. It was suggested that some TNBC patients may benefit from Pertuzumab in the future^[79]^. However, so far, only a few circRNA has been found to encode proteins and the functional significance of most circRNA-derived peptides is still unknown.

## THE ROLE OF CIRCULAR RNA IN TNBC PROGRESSION

### The effect of circRNA in TNBC proliferation, metastasis and invasion

Circular RNAs have a crucial role in the malignant progression of TNBC, including cell proliferation, metastasis, and invasion. The common metastases of TNBC include brain, liver, lung, and bone metastasis, which ultimately leads to a high mortality rate due to the presence of lesion metastasis in different parts and degrees. Many circRNAs are highly expressed in TNBC, such as circRRM2^[80]^, circ0000851^[81]^, circEZH2^[82]^, circ0042881^[83]^, and circZFAND6^[84]^ [Table 1]. A majority of them can enhance the miRNA sponge function, thus promoting the proliferation, migration, and invasion ability of TNBC, including circ0000851/miR-1183^[48]^, circEZH2/miR-217-5p^[82]^, circ0042881/miR-217^[83]^, and circZFAND6/miR-647^[84]^, thereby increasing tumor growth. In addition, a small portion of them can play a role in promoting tumor growth by encoding or binding proteins, including circCAPG/CAPG-171aa^[76]^, circEIF6/EIF6-224aa^[77]^, circKIF4A/EIF4A3^[70]^, CircSNX25/COPB1^[71]^, and circEIF3H/IGF2BP2^[73]^. There are also some circRNAs whose expression levels were low in TNBC, such as CircPTK2^[51]^, CircNR3C2^[58]^, circFBXW7^[11]^, circRNF10^[85]^, and circCCDC85A^[86]^. Overexpressing these circRNAs greatly reduces the ability of TNBC cells to grow and vice versa.

### The role of circRNA in TNBC metabolism

Tumor experts consider metabolic reprogramming as one of the six fundamental hallmarks of tumor progression^[87]^. New research indicates that circRNA also plays a crucial function in tumor metabolism^[88]^. Currently, there is considerable research on the role of circRNA in TNBC metabolism, mainly focusing on glucose metabolism^[89]^. Circ-PDCD11 has been found to promote the progression of TNBC by enhancing aerobic glycolysis^[55]^. In addition, circ_0039960 is upregulated in TNBC cells, and knockdown of circ_0039960 significantly inhibits lactate production and glucose uptake, hinders the glycolytic process, and thus inhibits the progression of TNBC^[90]^. Many enzymes involved in glucose metabolism are regulated by circRNAs, such as hexokinase2 (HK2) and lactate dehydrogenase A (LDHA). Dou *et al*. reported that circ_0008039 was upregulated in TNBC tissue and cells, and knockdown of circ_0008039 inhibited TNBC cell proliferation, migration, invasion, and glycolysis. Western blotting demonstrated that knockdown of circ_0008039 can decrease the expression level of HK2, which was a key rate-limiting enzyme of glycolysis^[91]^. CircRNF20 also regulates glycolysis by affecting HK2^[92]^. Circ-CSNK1G1 regulates the glycolysis level of TNBC by acting on lactate dehydrogenase (LDH) through miR-28-5p^[47]^. Besides, circRNA derived from Myc promotes the progression of TNBC by reprogramming fatty acid metabolism. However, there is currently relatively little research on circRNA in lipid metabolism and others. A future advance is anticipated in our understanding of these topics.

### The effect of circRNA in TNBC drug resistance

Currently, the primary method of treating TNBC in clinical settings involves chemotherapy, including doxorubicin, albumin paclitaxel, docetaxel, and platinum. Chemotherapy is the preferred option for TNBC and has benefited many TNBC patients. However, with the deepening of treatment, many patients have developed certain resistance to chemotherapy drugs, so their clinical use is still limited^[93]^. Chemo-resistant TNBC is a diverse and genetically unstable condition that presents a significant challenge to the application of personalized treatments^[94]^. Research has shown that circRNA plays an important role in drug resistance^[95,96]^, such as circ_0000199. It plays a role in TNBC chemo-resistance through the AKT3/miRNA pathway. This work validates the circ_ 0000199 resistance to four chemotherapy drugs, including cisplatin, adriamycin, paclitaxel, and gemcitabine. The MiR-206/miR-613 inhibitor blocked the negative effects of si-hsa_circ_0000199 on PI3K/Akt/mTOR signaling and the ability of TNBC cells to respond effectively to chemotherapy^[62]^. CircWAC is a circRNA that induces TNBC PTX resistance. It acted as a miR-142 sponge to control the PI3K/AKT signaling of TNBC cells and influence their chemosensitivity by reducing the repressive effect of miR-142 on its target WWP1^[57]^. Wang *et al*. found that circCREIT was downregulated in doxorubicin-resistant TNBC cells. Using a patient-derived organoid, they revealed that circCREIT overexpression greatly enhanced doxorubicin sensitivity of TNBC cells and demonstrated that this function is mainly achieved by stabilizing PKR^[97]^. In addition, circNCOR1 was shown to modulate TNBC radiotherapy resistance^[42]^, and circFGFR4 was associated with TNBC immunotherapy resistance^[43]^. Although basic research has demonstrated that circRNAs play an important role in TNBC resistance, relevant clinical trials have not yet been performed.

### The effect of circRNA in TNBC immune microenvironment

Many circRNAs have been discovered in ovarian cancer^[98]^, lung adenocarcinoma^[99]^, colorectal cancer^[100]^, esophageal cancer^[101]^, pancreatic cancer^[102]^, and oral squamous cell carcinoma^[103]^ in which they regulate tumor immune microenvironment. CircRNAs can induce programmed cell death^[104]^, and enhance PD-1/PD-L1 binding by increasing PD-1 expression, preventing T cell identification and triggering, thus causing immune escape of tumor cells. Moreover, circRNAs also regulate NK cells, macrophages, neutrophils, myeloid-derived inhibitory cells, and cancer-associated fibroblasts through complex pathways to affect tumor development. There are also some immune-related circRNAs in breast cancer. CircWWC3 induces repolarization of M1 macrophages to M2 by increasing the expression of IL-4 and PD-L1 that promotes the escape of immune cells from tumors and worsens the growth of TNBC. Breast cancer patients with high levels of circWWC3 or PD-L1 expression and high CD163-expressing macrophages are correlated with low overall survival (OS) and disease-free survival (DFS)^[105]^. Circ_0001142 was found to be highly expressed in BCs. It was packaged in exomes and released by endoplasmic reticulum stress which induces M1 macrophage repolarization and promotes TNBC progression through the PI3K/AKT pathway^[106]^.

### CircRNA and the clinical treatment of TNBC

Most tumor progression is due to a lack of early monitoring and examination of the body, and TNBC is no exception. Early detection and treatment can greatly improve the survival rate of TNBC patients. The structure of circRNA is stable and can be easily detected in blood, urine, saliva, and other biological tissues. Therefore, researchers are exploring the relationship between circRNA and tumor development and whether it can reflect the clinical prognosis of patients after treatment. Currently, many circRNAs have been found to be associated with the prediction, treatment, and prognosis of TNBC^[107]^. The relevant circRNAs identified in TNBC in recent years are summarized in Table 2. The vast majority of the circRNAs identified are pro-oncogenic, with elevated expression in TNBC. Only a few of them play an anti-tumor role and are downregulated in TNBC. Recent research found that they are all related to clinicopathological characteristics and OS or DFS. Among them, circCAPG^[76]^ (AUC 0.8723), circDNAJC11^[108]^ (AUC 0.658), circSEPT9^[112]^ (AUC 0.711), circRAD18^[116]^ (AUC 0.752), circTADA2A-E6^[117]^ (AUC 08554), and circAHNAK1^[119]^ (AUC 0.72) have a role as prognostic markers for TNBC treatment. In addition, circ 0072309^[124]^ can also be used as a biomarker, but there is currently no research on its relationship with clinicopathological characteristics and survival rate. Circ_0000851 is associated with Ki-67, tumor size, and lymph node metastasis, but the relationship to survival has yet to be elucidated^[81]^. It is possible that additional circRNAs will be discovered in the future, which could serve as prognostic markers and promising targets for the therapy of TNBC.

**Table 2:** CircRNAs associated with TNBC survival prognosis

**CircRNAs**	**Roles**	**TNBC patients**	**Expression**	**Survival**	**Clinicopathological characteristics**	**Ref.**
CircCAPG	Oncogenic	132	Up	OS	Tumor size, lymph node metastasis and TNM stage	[76]
CircEGFR	Oncogenic	38	Up	OS, DFS	No correlation	[39]
CircDNAJC11	Oncogenic	269	Up	OS	TNM stage	[108]
CircFGFR4	Oncogenic	60	Up	OS	Tumor size	[43]
CircCREIT	Antitumor	244	Down	OS	Pathological grade, lymph node metastasis and tumor size	[97]
CircTBC1D14	Oncogenic	237	Up	OS	Tumor size, Ki67 expression, lymph node metastasis, and distant metastasis	[109]
CircPTK2	Oncogenic	45	Up	OS	Not analyzed	[51]
Circ_0000977	Antitumor	82	Down	DFS	Tumor size and age	[110]
CircWAC	Oncogenic	90	Up	OS	No correlation	[57]
Circ_0044234	Antitumor	87	Down	DFS	Lymph node metastasis, Ki67 expression, and histological grade	[111]
CircERBB2	Oncogenic	82	Up	OS	TNM stage and lymph node metastasis	[56]
CircWHSC1	Oncogenic	65	Up	OS	Not analyzed	[38]
Circ 102229	Oncogenic	72	Up	OS	Tumor size, lymph node metastasis and TNM stage	[61]
CircNR3C2	Antitumor	60	Down	RFS	Not analyzed	[58]
CircSEPT9	Oncogenic	80	Up	OS	TNM stage	[112]
CircUSP42	Antitumor	30	Down	OS, DFS	Lymph node metastasis and clinical stage	[113]
CircGNB1	Oncogenic	222	Up	OS, DFS	Tumor size and clinical stage	[114]
Circ 0131242	Oncogenic	120	Up	OS	Tumor size and TNM stages	[115]
Circ-HER2	Oncogenic	59	Up	OS	Not analyzed	[79]
CircPGAP3	Oncogenic	86	Up	OS, DFS	Tumor size, lymph node metastasis and TNM stage	[64]
CircUBE2D2	Oncogenic	66	Up	OS	Not analyzed	[41]
CircRAD18	Oncogenic	126	Up	OS	T stage, tumor size and clinical stage	[116]
CircFBXW7	Antitumor	473	Down	OS, DFS	Tumor size and lymph node metastasis	[11]
CircTADA2A-E6	Antitumor	115	Up	OS, DFS	Lymphatic metastasis and clinical stage	[117]
Circ 069718	Oncogenic	35	Up	OS	TNM stage, lymph node metastasis	[118]
CircAHNAK1	Antitumor	136	Down	OS, DFS	Tumor size, lymph node metastasis, and TNM stage	[119]
CircITCH	Antitumor	91	Down	OS	Tumor size, lymph node metastasis and TNM stage	[120]
CircKIF4A	Oncogenic	240	Up	OS, DFS	Tumor size, lymph node metastasis and TNM stage	[121]
CircPLK1	Oncogenic	240	Up	OS, DFS	Tumor size, lymph node metastasis	[65]
CircANKS1B	Oncogenic	165	Up	OS	Lymph node metastasis and TNM stage	[122]
CircUBAP2	Oncogenic	78	Up	OS	Tumor size, lymph node metastasis and TNM stage	[52]
CircEPSTI1	Oncogenic	240	Up	OS, DFS	Tumor size, lymph node metastasis and TNM stage	[67]
CircGFRA1	Oncogenic	222	Up	OS, DFS	Tumor size, TNM stage, lymph node metastasis and histological grade	[123]
CircIFI30	Oncogenic	78	Up	OS	Age, histological grade and clinical stage	[63]

TNBC: Triple-negative breast cancer; OS: overall survival; DFS: disease-free survival.

## CONCLUSION

Breast cancer is a major life-threatening disease for women around the world and has to be examined in depth. As the most malignant type among them, TNBC deserves wider attention. Scientific and technological development has led to the gradual recognition of circRNA with special properties, which has become a popular area of research in recent years. CircRNA is widely expressed in organisms due to its structural stability. Recent studies indicated that circRNA is not a class of by-products, but a normal component of the body that plays an important regulatory role in a variety of diseases, including tumor. Accumulating evidence suggests that circRNA is related to the survival and prognosis of TNBC and can be used as a biomarker to predict outcomes of the treatment, which opens up another new direction for TNBC therapeutics in the clinic. However, many studies have shown that circRNAs are expressed with spatiotemporal dynamics even within the same tumor, which in turn poses limitations to its use as a therapeutic tool. Currently, there are still many issues that need further research regarding circRNAs: (1) although circRNAs play a significant role in TNBC tumorigenesis and development, our current understanding of their various functions is still limited; (2) in addition to studying the specific mechanisms of circRNA involvement in TNBC tumorigenesis and development, it is important to translate useful experimental findings into clinical products for the benefit of breast cancer patients; and (3) research on circRNA still focuses on sponge function, while the mechanism and function of circRNA as a translation template or binding protein are still relatively unknown. This review briefly explained the biosynthetic mechanism and functions of circRNA. We also described several major biological events in which circRNA is involved for the progression of TNBC, and summarized current information on circRNAs in relation to the survival prognosis of TNBC. This article did not explain how circRNA is degraded or about its regulatory relationship with parental genes. Additionally, the summarized biological events are not comprehensive, but representative of selected hot topics at present.

## DECLARATIONS

### Copyright

© The Author(s) 2024.
